# How *Mycobacterium tuberculosis* drug resistance has shaped anti-tubercular drug discovery

**DOI:** 10.3389/fcimb.2022.974101

**Published:** 2022-09-09

**Authors:** Amala Bhagwat, Aditi Deshpande, Tanya Parish

**Affiliations:** Center for Global Infectious Disease Research, Seattle Children’s Research Institute, Seattle, WA, United States

**Keywords:** Mycobacterium tuberculosis, antibiotic resistance, drug discovery, antibiotic tolerance, antibacterial

## Abstract

Drug resistance is an increasing problem for the treatment of tuberculosis. The prevalence of clinical isolates with pre-existing resistance needs to be considered in any drug discovery program. Non-specific mechanisms of resistance such as increased efflux or decreased permeability need to be considered both in developing individual drug candidates and when designing novel regimens. We review a number of different approaches to develop new analogs and drug combinations or improve efficacy of existing drugs that may overcome or delay the appearance of clinical resistance. We also discuss the need to fully characterize mechanisms of resistance and cross- resistance to existing drugs to ensure that novel drugs will be clinically effective.

## Introduction

Tuberculosis (TB) remains a major global health problem causing ~1.5 million deaths in 2020 (World Health Organization, 2021). Treatment of TB is complicated owing to the unique capacity of the causative bacterium (Mycobacterium tuberculosis), to survive within the human body. Although the bacilli are unable to replicate in acidic or hypoxic environments, such as found in the granuloma, M. tuberculosis can persist in these environments for lengthy periods. Latent TB infection (LTBI), in which the bacilli remain in the body without clinical symptoms poses a unique problem for the diagnosis and treatment of TB. In addition, drug resistance is a major problem which can result from both inherent and acquired resistance mechanisms. Thus TB control programs require both new drugs to overcome existing resistance. and rapid detection tests for drug resistance.

Although treatment of TB became possible with the discovery of streptomycin, there are few antibiotics available for modern use. The major frontline drugs for drug-susceptible TB, isoniazid and rifampicin have been supplemented with the recent addition of the new agents active against drug resistant TB, such as bedaquiline (a diarylquinolone), delaminid and pretomanid (nitroimidazoles) and repurposing of linezolid (an oxazolidinone), but the pipeline is still inadequate (Libardo et al., 2018; Oh et al., 2021). As for other antibiotics, resistance to streptomycin was observed very soon after its clinical use. Early clinical trials demonstrated the utility of combination regimens (Fox, 1979; Mitchison, 1985; Grosset, 1989) with the additional advantage that resistance to multiple agents is harder to acquire. However, given the length of time the frontline agents have been in use, a rise in drug resistance has been seen for rifampicin and isoniazid both singly and in combination (multi-drug resistance or MDR). Extensively drug resistant (XDR) strains are resistant to rifampicin, isoniazid and a fluoroquinolone.

Drug resistance in TB is largely mediated by chromosomal mutations, as there are no reports of horizontal gene transfer. However, there are multiple routes by which the bacilli can become resistant, not all of which involve mutation of the drug target. Mutations in the target which reduce or alleviate drug-binding do occur, as does mutation in the promoter leading to increased target expression. Drug inactivation, such as with the beta lactamases that degrade the beta lactams or modifying enzymes such as acetyl/methyl transferases are another resistance mechanism reported in M. tuberculosis (Zaunbrecher et al., 2009; Kurz and Bonomo, 2012). Other mechanisms which do not involve chromosomal mutation have been noted *in vitro* such as mistranslation of proteins leading to phenotypic resistance to rifampicin (Javid et al., 2014). In addition, changes in drug uptake or efflux are non-specific processes which can affect sensitivity to multiple drugs from the same or different chemical classes. M. tuberculosis has a lipid-rich outer cell wall which imparts intrinsic resistance by acting as a permeability barrier, and changes in cell wall composition can affect drug uptake (Jarlier and Nikaido, 1994). M. tuberculosis also has a variety of efflux systems which, if up-regulated, can lead to resistance (Rodrigues et al., 2017). For pro-drugs, such as isoniazid, occurrence of mutation in the activating enzymes can lead to drug resistance (Zhang et al., 1992; Zhang and Yew, 2009; Seifert et al., 2015). Given the variety of resistance mechanisms and the possibility of resistance to multiple drugs, an evaluation of the resistance mechanisms for new drugs is an important component of the drug discovery process, alongside the standard considerations of pre-existing resistance and resistance frequency.

The need for lengthy treatments (>6 months for drug sensitive TB) with multiple agents poses issues with adherence that can lead to the selection of resistant subpopulations during treatment. There is an urgent need to develop agents with new mechanisms that are not affected by pre-existing resistance, but also to shorten the duration of TB treatment to restrict the emergence of resistance. Thus, drug discovery for TB has been strongly shaped by the prevalence of existing resistance mechanisms, as well as the rate of resistance appearing in the clinic.

Drug discovery for TB has adopted several approaches which attempt to develop new agents to address the issue of pre-existing resistance and to the appearance of new resistance mechanisms. Several approaches have been used including: (i) Generating analogs which overcome resistance by binding to the target in a different fashion; (ii) Restoring sensitivity to antibiotics using booster or adjunct molecules; (iii) Using combinations to generate new regimens to minimize the appearance of resistance. We will review recent successes in these approaches and address some of the additional factors that should be considered when developing new agents (**Table 1**).

**Table 1:** Examples of resistance mechanisms to current TB drugs and approaches to overcome resistance (references in text).

Drug	Resistance mechanisms in TB	Strategies
Rifampicin	Mutation in *rpoB* hotspot region	New rifamycins New inhibitor classes
Isoniazid	Mutations in *katG* (lack of pro-drug activation) Mutations in *inhA* and promoter region (loss of binding to target)	Analogs which do not require activation Direct *inhA* inhibitors
Fluoro-quinolones	Mutations in *gyrA/B* (loss of binding to target)	Novel scaffolds Gyrase ATPase inhibitors GyrB inhibitors
Ethionamide	Mutations in *ethA* (lack of pro-drug activation)	Increase activation of pro-drug (disruption of EthR-DNA binding) Alternative mechanisms of prodrug activation (increased expression of MymA)
Beta-lactams	Beta lactamase inactivation	Beta- lactamase inhibitors Beta- lactamase resistant analogs
Aminoglycosides	Mutation in ribosomal RNA and protein *(rrs*, *rspL*) Inactivation by *eis* acetyl transferase	Eis inhibitors
All	Non-specific or intrinsic resistance e.g. increased efflux Antibiotic tolerance	Targeting efflux pumps e.g. EfpA

## Development of analogs of existing drugs

The standard drug regimen for TB has a high success rate for cure when used with drug sensitive strains. Thus, there has been a lot of emphasis on developing new analogs of these successful antibiotics, but which can overcome pre-existing resistance.

### New RNA polymerase inhibitors

The DNA-dependent RNA polymerase is the target of the frontline drug rifampicin. The majority of clinical resistance results from mutation in a hotspot of 81bp in the coding region of the target RpoB (Telenti et al., 1993; Mboowa et al., 2014; Zaw, 2018). Mutations in clinical isolates which result in changes in the hydrogen bonding and van der Waals interaction between RpoB and rifampicin are associated with clinical resistance (Li et al., 2021). Knowledge of the binding mechanism can be used to design novel derivatives which retain binding or to find molecules that bind to different sites on the RNA polymerase. Other members of the rifamycin class such as rifampin, rifapentine and rifabutin have the same pharmacophore which can result in cross-resistance (Alfarisi et al., 2017; Tiberi et al., 2017; Farhat et al., 2019). For example, H526C mutations lead to resistance to both rifampicin and rifabutin (Cavusoglu et al., 2004). Molecules with alternative binding sites/modes are of interest, for example fidaxomicin has *in vitro* activity against M. tuberculosis and a class of N-aroyl-N-aryl-phenyl-alaninamides were identified that bind to RNA polymerase and inhibit M. tuberculosis without cross-resistance (Lin et al., 2017; Kirsch et al., 2022). Development of these alternative RNA polymerase inhibitors could supplant rifampicin in a regimen and overcome clinical resistance.

### New InhA inhibitors

Isoniazid is one of the earliest anti-tubercular drugs and works *via* inhibition of InhA, a component of FAS-II (fatty acid synthase) involved in synthesis of mycolic acids, key cell wall components. Isoniazid is a prodrug which is activated intracellularly by the KatG catalase-peroxidase (Zhang et al., 1992). The activated molecule forms an adduct with NAD(H) at the active site of the enzyme (Banerjee et al., 1994; Rawat et al., 2003). There are multiple routes to isoniazid resistance: (i) mutations in KatG (most commonly S315T) which reduce its enzymatic activity leading to lack of activation of isoniazid; (ii) mutations in the target InhA which lead to lack of binding (Tseng et al., 2015); and (iii) mutations in the promoter region which lead to increased expression of InhA (Seifert et al., 2015). A combination of mutations in the promoter and InhA are often seen clinically with highly resistant strains (Seifert et al., 2015).

In order to generate analogs which overcome resistance, the development of direct InhA inhibitors which do not require activation shows promise. Early work on triclosan and its derivatives confirmed that it was possible to develop alternative inhibitors for InhA (Armstrong et al., 2020; Rodriguez et al., 2020; Chetty et al., 2021) and multiple scaffolds, as well as a natural product, have been identified which can inhibit InhA (Pan and Tonge, 2012). These newer analogs generally do not require activation and bind directly to InhA, thus they can overcome resistance due to KatG and InhA mutation. A series of hydroxy-pyridones which do not require activation are active against common isoniazid resistant clinical strains (Manjunatha and Smith, 2015), as are several classes of thiadiazoles which inhibit InhA directly (Šink et al., 2015; Martínez-Hoyos et al., 2016). In addition, diazaborines which do not require activation or binding to NADH have been developed (Xia et al., 2018) which are active against isoniazid resistant clinical isolates. These also demonstrate good activity against both replicating and non-replicating bacteria (Flint et al., 2020) suggesting they might be able both to overcome pre-existing resistance and shorten therapy by eliminating persistent organisms. The natural product pyridomycin also targets InhA, as a competitive binder for NADH and is active against most clinically-resistant isolates (Hartkoorn et al., 2012). In addition to overcoming existing resistance new analogs which do not require activation would have a lower frequency of resistance, so drug resistance in the clinic would likely appear more slowly. This has been demonstrated in animal models, where the diazborine AN12855 had a lower frequency of resistance in mice as compared to INH (Robertson et al., 2019).

### New gyrase inhibitors

Fluoroquinolones are broad-spectrum antibiotics with bactericidal activity which target DNA gyrase and DNA topoisomerase. In M. tuberculosis, DNA gyrase is the sole target, since it lacks the topoisomerase (Nagaraja et al., 2017; Aubry et al., 2004) Fluoroquinolones are attractive since they have activity against replicating, non-replicating and intracellular M. tuberculosis. Resistance to fluoroquinolones in M. tuberculosis is due to mutations in DNA gyrase (Avalos et al., 2015); high level resistance is generally conferred by mutation in the GyrA subunit in the quinolone resistance determining region covering codons 74-113 (Soudani et al., 2010; Singh et al., 2015; Singh et al., 2021; Chaoui et al., 2018). A single mutation can lead to resistance to the entire class of fluoroquinolones, therefore novel agents with different binding modes would be useful.

One approach to overcome resistance encoded by gyrA mutations, has been to identify novel scaffolds that target gyrase *in vitro*. Examples include the naphthyridone/aminopiperidines (Gibson et al., 2019) and alkoxytriazoloquinolones (Carta et al., 2019). The spiropyrimidinetrione series has activity against M. tuberculosis strains with mutations in gyrase suggesting a potential to overcome fluoroquinolone resistance (Basarab et al., 2022). In addition, the possibility of targeting GyrB has been addressed (Stokes et al., 2020); for example, the aminopyrazinamides and 2-amino-5-phenylthiophene-3-carboxamide (Shirude et al., 2013; Saxena et al., 2015) which target GyrB have good potency *in vitro*.

## Restoring/improving the activity of existing agents

M. tuberculosis is intrinsically resistant (or can become resistant) to several classes of antibiotics *via* expression of drug-metabolizing enzymes. The bacilli also have efflux systems which can minimize intracellular accumulation and target engagement. Examples of efforts to overcome these intrinsic resistance mechanisms are described below and may lead to new strategies for prolonging the useful life of an antibiotic and/or reducing the required dose.

Ethionamide (ETH) is a prodrug which is activated by M. tuberculosis EthA to form an NAD-adduct which binds to InhA and inhibits mycolic acid synthesis (similar to the mode of action of isoniazid) (Vannelli et al., 2002). EthA, a flavin mono-oxygenase, is negatively regulated by the transcriptional regulator EthR. Inhibition of EthR leads to up-regulation of EthA which increases the activity of ETH. Small molecule inhibitors which disrupt EthR-DNA binding are able to “boost” the activity of ETH significantly, leading to activity *in vivo* at reduced doses (Willand et al., 2009) and could improve the clinical utility of ETH.

Ethionamide efficacy can also be “boosted” by the N-acylated 4-phenylpiperidine series (Flipo et al., 2022). These molecules interact with the VirS transcriptional regulator leading to the increased expression of MymA, a monooxygenase which activates ethionamide. This approach was successful in overcoming ethionamide resistance due to EthA mutations *in vitro* and in an animal model of infection.

M. tuberculosis is intrinsically resistant to beta lactams due to the expression of beta lactamase, but this can be reversed by the addition of beta lactamase inhibitors. For example, meropenem is highly effective *in vitro* when combined with clavulanate, as are the cephalosporins (Hugonnet et al., 2009; Ramón-García et al., 2016). The clinical effectiveness of meropenem is less clear, due to tolerability issues (De Jager et al., 2022), but this has led to an increased effort to find new beta lactams (Gold et al., 2022).

The M. tuberculosis acetyltransferase Eis can modify aminoglycosides thereby inactivating them (Willby et al., 2016). Increased expression of the enzyme leads to kanamycin resistance (Zaunbrecher et al., 2009) whereas inactivation of Eis restores kanamycin sensitivity. Several series of Eis inhibitors have been identified Willby et al., 2016; Punetha et al., 2020; (Punetha et al., 2021). Although kanamycin is unlikely to be used clinically since it is not orally available, this approach does lend proof of concept to the idea that targeting antibiotic modifying enzymes can overcome intrinsic resistance.

Drug efflux is a common mechanism of intrinsic resistance in many bacterial species, and M. tuberculosis encodes many efflux systems (Louw et al., 2009; Rodrigues et al., 2017). Differences in the expression or activity of efflux pumps in clinical isolates has been linked to resistance and over-expression of several systems (mmr, mmpL7, Rv1285c, p55 and efpA) was noted in response to drug treatment (Machado et al., 2017). Increased efflux is linked to antibiotic tolerance and the development of drug resistance (Pasipanodya and Gumbo, 2011). Thus targeting efflux and/or specific efflux pumps has been proposed as a way to improve efficacy of drugs and reduce resistance, although inhibiting efflux non-specifically can have issues with selectivity and/or toxicity (Rodrigues et al., 2020). Inhibitors of the EfpA efflux pump were recently identified (Johnson et al., 2019). EfpA plays a role in antibiotic tolerance in mycobacteria since its over-expression led to decreased uptake of several antibiotics including moxifloxacin (Rai and Mehra, 2021). Thus inhibitors of this system might have a dual function, since inhibition of EfpA inhibits growth, but could also prevent induction of tolerance.

## Using combinations to reduce resistance

### Combination regimens

The general consensus in anti-bacterial drug discovery is that the appearance of resistance occurs within a decade of widespread use for any new drug. If resistance can be delayed, this prolongs the useful life of a new drug. Standard TB therapy consists of a four drug regimen, partly because the drugs are insufficient on their own, but also because the combination of drugs can be very effective in delaying the appearance of resistance. Since the majority of target-based resistance is due to chromosomal mutations in M. tuberculosis, combining drugs is an effective way to reduce the frequency of resistance (since bacteria would need to be resistant to more than one agent simultaneously at the outset). Thus the development of new regimens, rather than individual drugs, is standard practice for TB. However, there are still additional considerations for generating the best regimens. In particular, the resistance mechanism(s) for each drug in the regimen needs to be different. Combining drugs which hit different targets is not sufficient to prevent cross-resistance, due to the possibility of non-specific resistance mechanisms. Recent experience using monotherapy with bedaquiline has demonstrated that low level clinical resistance can appear quickly and that it can involve non-specific mechanisms, such as increased drug efflux (see below). Therefore considering the susceptibility of novel agents to common resistance mechanisms is important.

### Dual targeting molecules

An alternative approach to developing individual agents for a combination regimen is to develop agents that simultaneously inhibit more than one target. This has been proposed both for targets from the same family as well as for targets with different active sites. For example, uridine derivatives that target multiple Mur enzymes (involved in the same pathway of peptidoglycan synthesis) have been identified (Kumari et al., 2022), as well as “ionized non-classical antifolates” that target both dihydrofolate reductase and thymidylate synthase (Hajian et al., 2019); the thiophene carboxamide IMB-T130 which targets both tyrosyl-tRNA synthetase and dehydroquinate synthase (Zhu et al., 2015; Zhu et al., 2018); and SQ109 which targets both MmpL3 and respiration (Kai et al., 2014; Li et al., 2014, 3). Although this approach could be useful to reduce the frequency of resistance to a single agent it may pose difficulties with respect to optimization for multiple targets, dosing and pharmacokinetics due to variation in the expression level, essentiality and vulnerability of the targets.

## Overcoming drug tolerance and eradicating persistent organisms

Antibiotic tolerance is assumed to be one of the major reasons that TB therapy takes many months; the persistence of genotypically sensitive, but phenotypically resistant bacilli may be a consequence of the physiological state(s) induced by host-induced stresses such as acidic pH, hypoxia or nutrient starvation (Mandal et al., 2019). Antibiotic tolerance is a precursor to the appearance of drug resistant bacilli since it allows for extended periods of survival in fluctuating concentrations of antimicrobial agents. Therefore, developing novel drugs that can shorten therapy would be a major advance in preventing or delaying the appearance of resistant isolates in the clinic. A number of groups have conducted high throughput screens to identify agents which target non-replicating organisms induced by different *in vitro* stresses including hypoxia, low pH, nitric oxide, cholesterol and nutrient starvation, as well as multi-stress models combining these (reviewed in (Parish, 2020). Such screens have identified numerous scaffolds for investigation. The most advanced compound GSK286, which was identified in a macrophage screen, targets cholesterol metabolism and is currently in a Phase I clinical trial (GlaxoSmithKline, 2022; Nuermberger et al., 2022).

## The impact of broad resistance mechanisms on early drug discovery

Bedaquiline, a member of class of diarylquinolines, inhibits ATP generation by binding to the C subunit of F0-F1 of the ATP synthase. High level resistance results from mutations in AtpE which reduce binding affinity. However, other mechanisms of resistance are found including mutations in the transcriptional repressor Rv0678 (efflux pump regulator) (Andries et al., 2014) and pepQ (Hartkoorn et al., 2014; Almeida et al., 2016). Mutations in Rv0678 lead to upregulation of the MmpL5/MmpS5 efflux system and increased efflux of the drug. Since this system also effluxes other drug classes, including azoles, clofazimine and macozinone (Hartkoorn et al., 2014; Chen et al., 2022; Guo et al., 2022), the appearance of these mutations in clinical isolates will lead to cross-resistance to multiple antimycobacterial classes. Similarly mutations in pepQ result in resistance to other agents such as macozinone (Chen et al., 2022; Guo et al., 2022), This underscores the need to determine mechanisms of resistance for new agents for both low-level and high-level resistance. In addition, since Rv0678 mutations occur in clinical isolates (Andries et al., 2014), mutant strains with these SNPs should form part of any clinical isolate panel used for routine testing during drug discovery.

## Determining mechanisms of resistance during the discovery phase

Phenotypic screening has been very successful in identifying new scaffolds for development. The disadvantage of whole cell screens is that the target is not known from the outset, so much effort has been put into developing target identification and validation methods. One of the most commonly-used methods is to isolate resistant mutants and characterize the chromosomal mutations. This can provide valuable information about potential target(s) and insight into the mechanism(s) of resistance. In these studies, the major focus has been on determining the frequency of resistance and of identifying mutations that lead to high level resistance.

Identification of the target and mutations that affect inhibitor binding can be invaluable in designing new analogs. However, there can be a disconnect between the mutations found *in vitro* and those that arise *in vivo* during treatment. For example, complete loss of KatG activity results in attenuation of M. tuberculosis but is the most common mechanism of isoniazid resistance isolated in vitro. In contrast, mutations which reduce the activity of KatG are more often seen *in vivo* (Vilchèze and Jacobs, 2014). Similarly the spectrum of mutations seem for linezolid are different *in vitro* from *in vivo* (Lee et al., 2012; McNeil et al., 2017). In clinical isolates of M. tuberculosis, resistance-conferring mutations are often accompanied by compensatory mutations that increase the overall fitness of the pathogen by restoring the activity of the drug target (Alame Emane et al., 2021). High level resistance can result from multiple mutations in the drug target which may affect binding and/or activity. As noted above, non-specific resistance mechanisms can also lead to low level resistance. Thus identifying mechanisms of resistance that arise using both *in vitro* and *in vivo* using relevant infection models are important to include in drug discovery efforts, as well as testing against a large panel of isogenic strains and clinical isolates.

## Conclusion

Drug discovery for tuberculosis is notoriously difficult due to the nature of the bacterium and the pathology of the disease. The existence of resistance in clinical isolates and the probability of resistance developing to new agents in the clinic poses further restraints on drug development. Several approaches to deal with the prevalence of clinically-resistant isolates have been tried including the development of analogs of existing frontline drugs and potentiation of the efficacy of existing drugs. The development of novel combination regimens aims to reduce the appearance of resistance. In practical terms, during the development of novel anti-microbials, a wide range of clinical isolates carrying known resistance-associated mutations should form part of a screening panel. Ideally, such a panel would also include strains with decreased permeability and increased efflux. In addition, a full characterization of mutations that lead to low level and high-level resistance *in vitro* and *in vivo* should form part of the characterization of any drug candidate.

## Author contributions

All authors wrote and edited the manuscript. All authors contributed to the article and approved the submitted version.

## Funding

This work was funded by NIAID of the National Institutes of Health under award number R01AI129360 and the Department of Defense Office of the Congressionally Directed Medical Research Programs under award number PR191269.

## Conflict of interest

The authors declare that the research was conducted in the absence of any commercial or financial relationships that could be construed as a potential conflict of interest.

## Publisher’s note

All claims expressed in this article are solely those of the authors and do not necessarily represent those of their affiliated organizations, or those of the publisher, the editors and the reviewers. Any product that may be evaluated in this article, or claim that may be made by its manufacturer, is not guaranteed or endorsed by the publisher.
